# Dataset of Fourier transform-infrared coupled with chemometric analysis used to distinguish accessions of *Garcinia mangostana* L. in Peninsular Malaysia

**DOI:** 10.1016/j.dib.2016.04.062

**Published:** 2016-05-04

**Authors:** Sri A’jilah Samsir, Hamidun Bunawan, Choong Chee Yen, Normah Mohd Noor

**Affiliations:** a Institute of Systems Biology, Universiti Kebangsaan Malaysia, 43600 Bangi, Selangor, Malaysia; b School of Environment and Nature Resource Science, Faculty Science and Technology, Universiti Kebangsaan Malaysia, 43600 Bangi, Selangor, Malaysia

**Keywords:** Apomictic, Mangosteen, Fourier Transformed-Infrared, Peninsular Malaysia

## Abstract

In this dataset, we distinguish 15 accessions of *Garcinia mangostana* from Peninsular Malaysia using Fourier transform-infrared spectroscopy coupled with chemometric analysis. We found that the position and intensity of characteristic peaks at 3600–3100 cm^−^^1^ in IR spectra allowed discrimination of *G. mangostana* from different locations. Further principal component analysis (PCA) of all the accessions suggests the two main clusters were formed: samples from Johor, Melaka, and Negeri Sembilan (South) were clustered together in one group while samples from Perak, Kedah, Penang, Selangor, Kelantan, and Terengganu (North and East Coast) were in another clustered group.

**Specifications Table**

**Table:** 

Subject area	*Biology*
More specific subject area	*Plant Sciences*
Type of data	*Figure; Table*
How data was acquired	*Fourier Transform-Infrared spectroscopy (Perkin-Elmer Frontier TM with a spectrum software version 10.3)*
Data format	*Analyzed*
Experimental factors	*Leaf of* Garcinia mangostana *from 15 different locations throughout Peninsular Malaysia were analysed using Fourier Transform-Infrared (FTIR) spectroscopy coupled with chemometric analysis.*
Experimental features	*Due to its reproductive manner,* Garcinia mangostana *trees are essentially clonal, FTIR coupled with chemometric analysis was used to primarily discriminate and to identify functional groups or chemical bonds in several accessions of* Garcinia mangostana *in Peninsular Malaysia. This approach is the first fingerprint identification for this apomictic clone plant.*
Data source location	*Peninsular Malaysia*
Data accessibility	*The data is available with this article.*

## 1 Value of the data

• Fourier transform-infrared (FTIR) is a fast, effective and non-destructive procedure to provide unique fingerprints without any sample pretreatment [1], [2].
• As an obligate apomictic plant, the genetic diversity of *Garcinia mangostana* is relatively narrow [3], [4]. FTIR spectroscopic data in combination with multivariate statistical analysis were performed to discriminate *G. mangostana* in Peninsular Malaysia.
• FTIR and multivariate analysis are able to separate *G. mangostana* in Peninsular Malaysia into two clusters.

## 2 Data

**Fig. 1:**
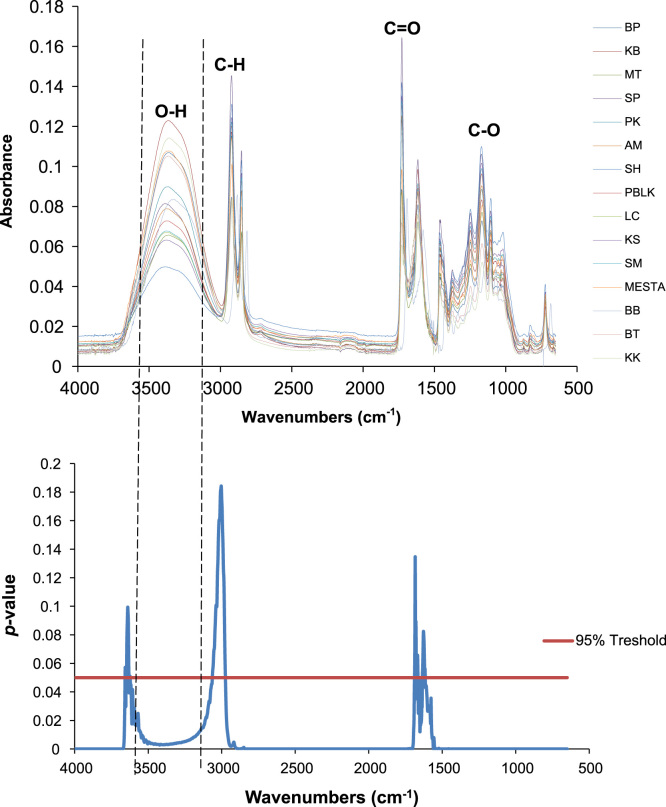
Four major functional groups present in leaves of *Garcinia mangostana* and their *p*-value. BP: Kg. Sungai Rusa, Balik Pulau; KB: Kampung Belukar, Tumpat; MT: MARDI Terengganu; SP: Kg. Tandap Batu, Sungai Petani; PK:Pengkalan Kubor, Kelantan; AM: Kg. Air Melintang Kota; SH: Kg. Senama Hilir, Rembau; PBLK: Pengkalan Balak, Alor Gajah; LC: Lubuk Cina, Melaka; KS: Kg. Solok, Tangkak; SM: Sungai Mati, Muar; MESTA: Rumah Tumbuhan, UKM; BB: Bukit Besi; BT: MARDI Bukit Tangga; KK: MARDI Kuala Kangsar.

**Fig. 2:**
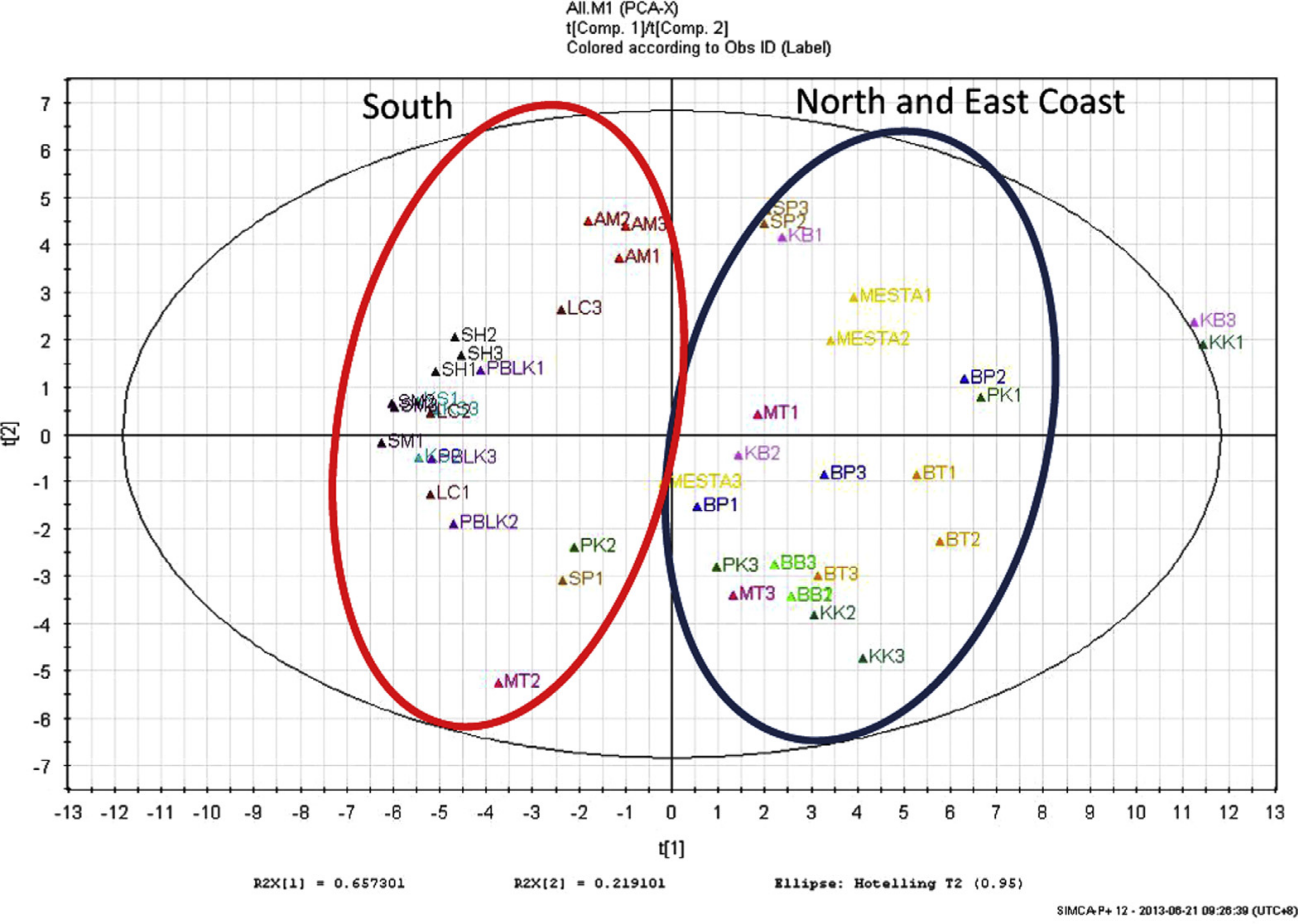
PCA analysis showed two major clustered groups: South, and North and East Coast region.

FTIR spectra (4000–650 cm^−1^) identified four major functional groups (O–H, C–H, C=O, and C–O) in the leaves of *G. mangostana* (Fig. 1) from 15 different sample locations in Peninsular Malaysia (Table 1). Principal component analysis (PCA) revealed two major clustering groups: samples from Johor, Melaka, and Negeri Sembilan (South) were clustered together in one group while samples from Perak, Kedah, Penang, Selangor, Kelantan, and Terengganu (North and East Coast) were in another clustered group (Fig. 2).

## 3 Experimental design, materials and methods

### 3.1 FTIR absorption spectra

**Fig. 3:**
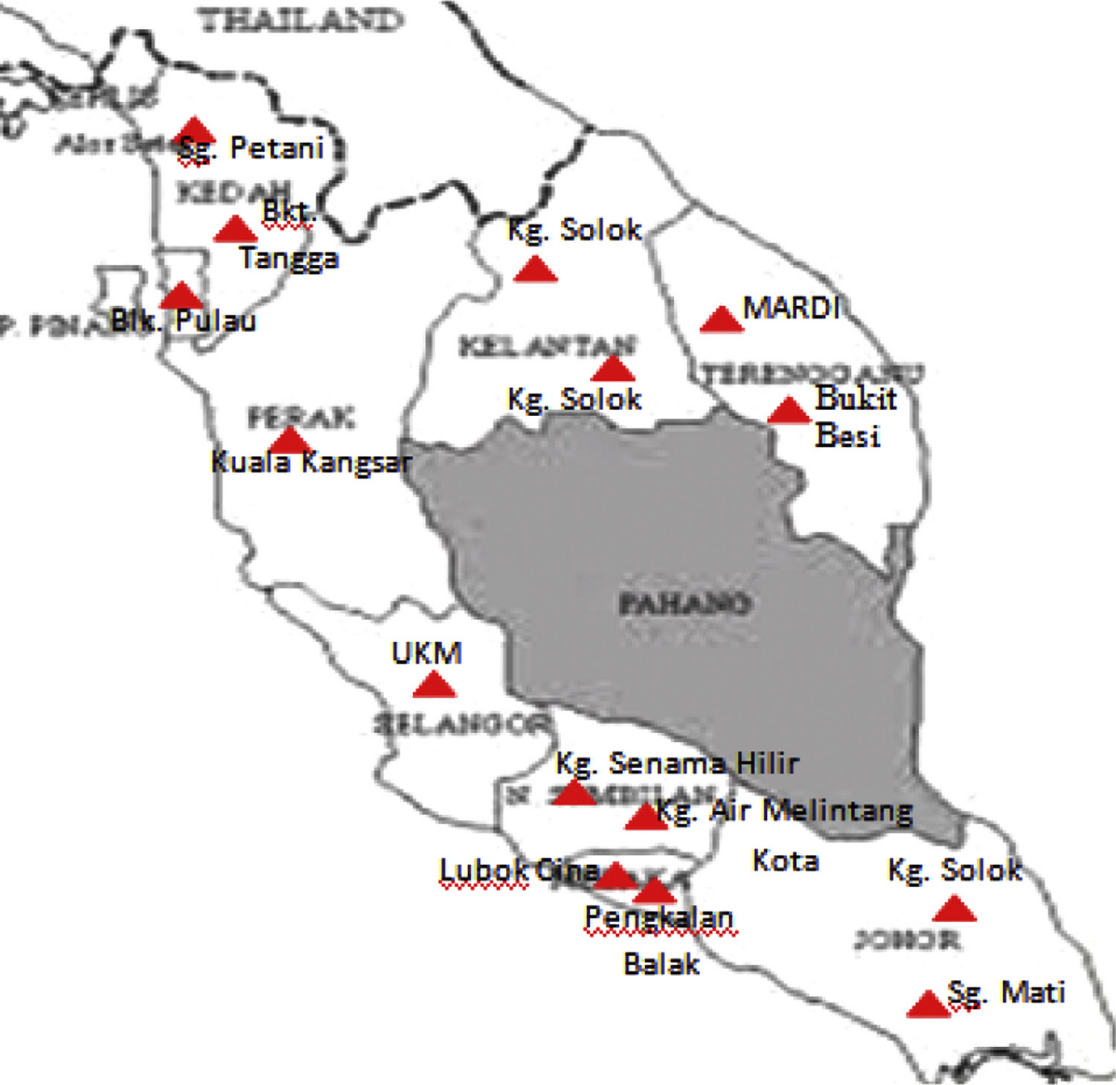
Map of 15 different locations where accessions of *G. Mangostana* were collected.

Leaves of *G. mangostana* from 15 different locations throughout Peninsular Malaysia (Fig. 3) were collected and the GPS location were recorded (Table 1). FTIR analysis was conducted using Perkin-Elmer Frontier^TM^ with spectrum software version 10.3 (Perkin-Elmer, USA) for sample discrimination. Samples were placed on the top surface of the crystal and the gripper plate positioned gently on top to avoid damage to the crystal. The crystal was protected from scratches to ensure even contact with the sample and avoid undue effect on penetration depth that could confer aberrant results. IR spectra were recorded in the 4000–650 cm^−1^ range. All analyses were conducted with three biological replicates, each with three technical replicates, and the samples were randomly ordered to avoid bias. Data sets were baseline-corrected and area-normalized before statistical analysis.

### 3.2 Statistical analysis

Principal component analysis (PCA) was conducted using SIMCA-P software to discriminate and classify the samples. Differences between combined data of different locations were analyzed using student׳s *t-*test analysis in SPSS version 12.0.1 software. A value of *p*<0.05 was considered to be significant.

## Conflict of Interest

The authors declare there is no conflict of interest.

## Figures and Tables

**Table 1:** 15 different locations for sampling of *G. Mangostana*.

**Locations**	**Latitude (N)**	**Longitude (E)**
Bukit Besi, Terengganu	N: 4° 56׳ 51.2"	E: 103° 10׳ 22.5"
MARDI Terengganu	N: 4° 57׳ 14.3"	E: 103° 10׳ 8.7"
Pengkalan Kubor, Kelantan	N: 6° 10׳ 36.3"	E: 102° 07׳ 28.5"
Kg. Belukar, Tumpat	N: 6° 12׳ 16.9"	E: 102° 06׳ 31.9"
Rumah Tumbuhan, UKM	N: 2° 55׳ 13"	E: 101° 47׳ 2"
Kg. Sungai Rusa, Balik Pulau	N: 5° 20׳ 43.0"	E: 100° 13׳ 45.9"
Kg. Tandop Batu, Sg. Petani	N: 5° 43׳ 19.9"	E: 100° 24׳ 54.1"
MARDI Bukit Tangga	N: 6° 29׳ 7.2"	E: 100° 28׳ 58.6"
MARDI Kuala Kangsar	N: 4° 45׳ 51.1"	E: 100° 54׳ 21.8"
Kg. Senama Hilir, Rembau	N: 2° 34׳ 21.0"	E: 102° 05׳ 49.6"
Kg. Air Melintang Kota	N: 2° 30׳ 6.4"	E: 102° 06׳ 39.4"
Lubok Cina, Melaka	N: 2° 27׳ 40.3"	E: 102° 04׳ 4.6"
Pengkalan Balak, Alor Gajah	N: 2° 22׳ 54.5"	E: 102° 13׳ 5.7"
Kg. Solok, Tangkak	N: 2° 15׳ 33.4"	E: 102° 32׳ 36.8"
Sg. Mati, Muar	N: 2° 07׳ 36.0"	E: 102° 33׳ 27.6"
